# 7-Bromo-1-(3-fluoro­phenyl­sulfon­yl)-2-methyl­naphtho­[2,1-*b*]furan

**DOI:** 10.1107/S1600536812040470

**Published:** 2012-09-29

**Authors:** Hong Dae Choi, Pil Ja Seo, Uk Lee

**Affiliations:** a Department of Chemistry, Dongeui University, San 24 Kaya-dong, Busanjin-gu, Busan 614-714, Republic of Korea; b Department of Chemistry, Pukyong National University, 599-1 Daeyeon 3-dong, Nam-gu, Busan 608-737, Republic of Korea

## Abstract

In the title compound, C_19_H_12_BrFO_3_S, the 3-fluoro­phenyl ring makes a dihedral angle of 80.85 (5)° with the mean plane [r.m.s. deviation = 0.009 (2)Å] of the naphtho­furan fragment. In the crystal, mol­ecules are linked by slipped π–π inter­actions between the furan and the outer benzene rings of neighbouring mol­ecules [centroid–centroid distance = 3.756 (3) Å and slippage of 1.189 (3) Å].

## Related literature

For background information and the crystal structures of related compounds, see: Choi *et al.* (2008 ▶, 2011 ▶).
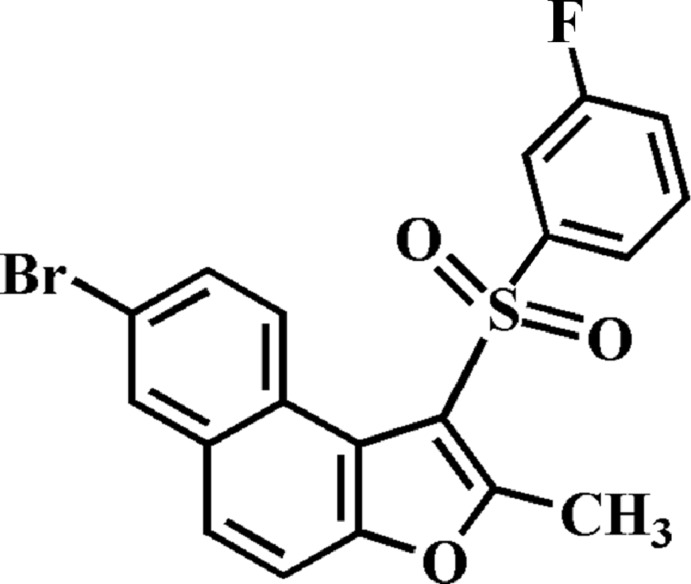


## Experimental

### 

#### Crystal data

C_19_H_12_BrFO_3_S
*M* *_r_* = 419.26
Triclinic, 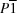

*a* = 7.7141 (2) Å
*b* = 8.1619 (2) Å
*c* = 13.4046 (4) Å
α = 74.277 (2)°
β = 86.410 (2)°
γ = 89.044 (2)°
*V* = 810.80 (4) Å^3^
*Z* = 2
Mo *K*α radiation
μ = 2.69 mm^−1^
*T* = 173 K
0.28 × 0.24 × 0.23 mm

#### Data collection

Bruker SMART APEX II CCD diffractometer
Absorption correction: multi-scan (*SADABS*; Bruker, 2009 ▶) *T*_min_ = 0.573, *T*_max_ = 0.746
15010 measured reflections
4031 independent reflections
3483 reflections with *I* > 2σ(*I*)
*R*_int_ = 0.047

#### Refinement

*R*[*F* ^2^ > 2σ(*F* ^2^)] = 0.030
*wR*(*F* ^2^) = 0.075
*S* = 1.06
4031 reflections
227 parameters
H-atom parameters constrained
Δρ_max_ = 0.49 e Å^−3^
Δρ_min_ = −0.40 e Å^−3^

### 

Data collection: *APEX2* (Bruker, 2009 ▶); cell refinement: *SAINT* (Bruker, 2009 ▶); data reduction: *SAINT*; program(s) used to solve structure: *SHELXS97* (Sheldrick, 2008 ▶); program(s) used to refine structure: *SHELXL97* (Sheldrick, 2008 ▶); molecular graphics: *ORTEP-3* (Farrugia, 2012 ▶) and *DIAMOND* (Brandenburg, 1998 ▶); software used to prepare material for publication: *SHELXL97*.

## Supplementary Material

Crystal structure: contains datablock(s) I. DOI: 10.1107/S1600536812040470/rk2381sup1.cif

Structure factors: contains datablock(s) I. DOI: 10.1107/S1600536812040470/rk2381Isup2.hkl

Supplementary material file. DOI: 10.1107/S1600536812040470/rk2381Isup3.cml

Additional supplementary materials:  crystallographic information; 3D view; checkCIF report

## Figures and Tables

**Fig. 1.**
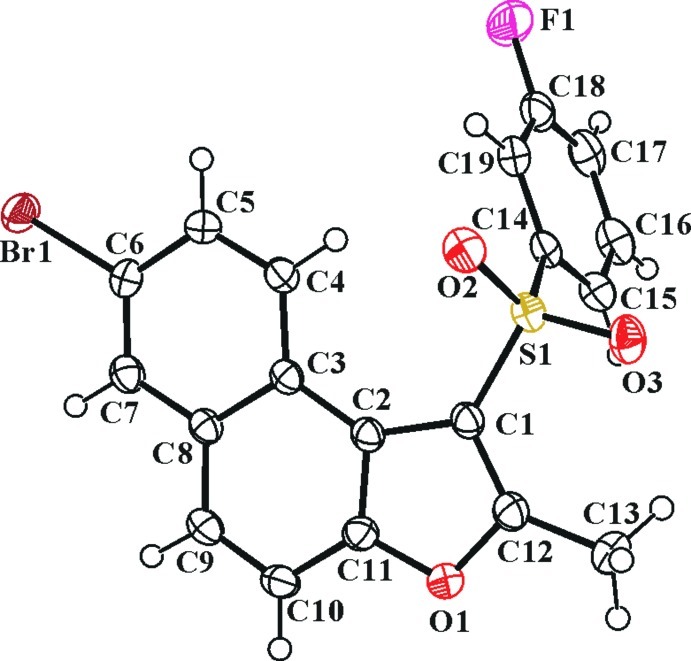
The molecular structure of the title compound with the atom numbering scheme. Displacement ellipsoids are drawn at the 50% probability level. H atoms are presented as small spheres of arbitrary radius.

**Fig. 2.**
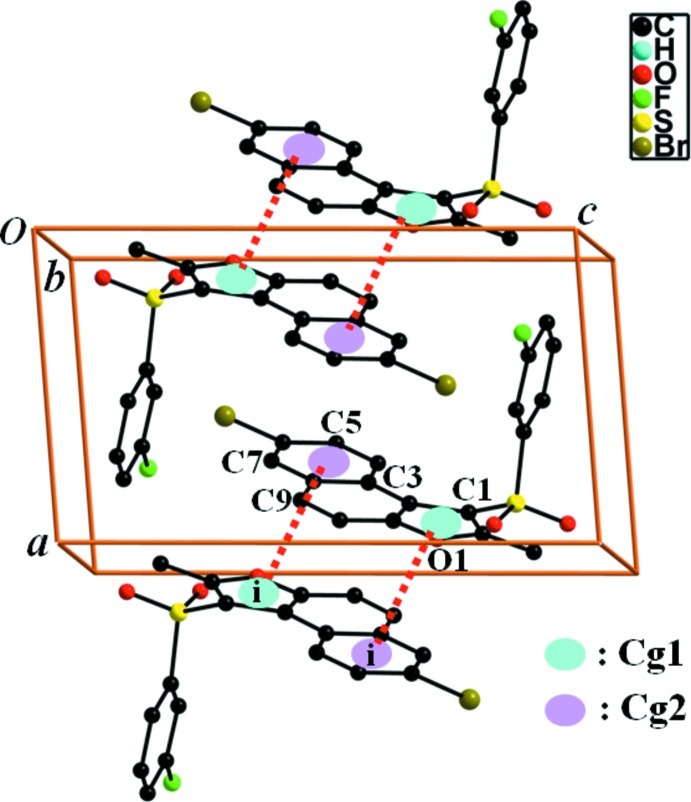
A view of π···π interactions (dotted lines) in the crystal structure of the title compound. All H atoms were omitted for clarity. Symmetry code: (i) -x+2, -y+1, -z+1.

## supplementary crystallographic information

### Comment

As a part of our ongoing study of 7-bromo-2-methylnaphtho[2,1-b]furan derivatives containing 1-(4-methylphenylsulfonyl) (Choi *et al.*, 2008) and 1-(4-fluorophenylsulfonyl) (Choi *et al.*, 2011) substituents, we report herein the crystal structure of the title compound.

In the title molecule (Fig. 1), the naphthofuran unit is essentially planar, with a mean deviation of 0.009 (2)Å from the least-squares plane defined by the thirteen constituent atoms. The dihedral angle formed by the mean plane of the naphthofuran ring and the 3-fluorophenyl ring is 80.85 (5)°. In the crystal structure (Fig. 2), molecules are connected by slipped π···π interactions between the furan and outer benzene rings of neighbouring molecules, with a Cg1···Cg2^i^ distance of 3.756 (3)Å and an interplanar distance of 3.563 (3)Å resulting in a slippage of 1.189 (3)Å (Cg1 and Cg2 are the centroids of the C1/C2/C11/O1/C12 furan ring and the C3–C8 benzene ring, respectively). Symmetry code: (i) -*x*+2, -*y*+1, -*z*+1.

### Experimental

The 77% 3-chloroperoxybenzoic acid (336 mg, 1.5 mmol) was added in small portions to a stirred solution of 7-bromo-1-(3-fluorophenylsulfanyl)-2-methylnaphtho[2,1-*b*]furan (271 mg, 0.7 mmol) in dichloromethane (40 mL) at 273 K. After being stirred at room temperature for 10 h, the mixture was washed with saturated sodium bicarbonate solution and the organic layer was separated, dried over magnesium sulfate, filtered and concentrated at reduced pressure. The residue was purified by column chromatography (benzene) to afford the title compound as a colourless solid - yield 67%, m.p. 472–473 K; *R*f = 0.68 (benzene). Single crystals suitable for X-ray diffraction were prepared by slow evaporation of a solution of the title compound in ethyl acetate at room temperature.

### Refinement

All H atoms were positioned geometrically and refined using a riding model, with C—H = 0.95Å for aryl and 0.98Å for methyl H atoms. *U*_iso_(H) = 1.2*U*_eq_(C) for aryl H atoms and *U*_iso_(H) = 1.5*U*_eq_(C) for methyl H atoms. The positions of methyl hydrogens were optimized rotationally.

### Crystal data

**Table:**

C_19_H_12_BrFO_3_S	*Z* = 2
*M**_r_* = 419.26	*F*(000) = 420
Triclinic, *P*1	*D*_x_ = 1.717 Mg m^−^^3^
Hall symbol: -P 1	Melting point = 472–473 K
*a* = 7.7141 (2) Å	Mo *K*α radiation, λ = 0.71073 Å
*b* = 8.1619 (2) Å	Cell parameters from 7012 reflections
*c* = 13.4046 (4) Å	θ = 2.6–28.3°
α = 74.277 (2)°	µ = 2.69 mm^−^^1^
β = 86.410 (2)°	*T* = 173 K
γ = 89.044 (2)°	Block, colourless
*V* = 810.80 (4) Å^3^	0.28 × 0.24 × 0.23 mm

### Data collection

**Table:**

Bruker SMART APEX II CCD diffractometer	4031 independent reflections
Radiation source: rotating anode	3483 reflections with *I* > 2σ(*I*)
Graphite multilayer monochromator	*R*_int_ = 0.047
Detector resolution: 10.0 pixels mm^-1^	θ_max_ = 28.3°, θ_min_ = 1.6°
φ– and ω–scans	*h* = −10→10
Absorption correction: multi-scan (*SADABS*; Bruker, 2009)	*k* = −10→10
*T*_min_ = 0.573, *T*_max_ = 0.746	*l* = −17→16
15010 measured reflections	

### Refinement

**Table:**

Refinement on *F*^2^	Primary atom site location: structure-invariant direct methods
Least-squares matrix: full	Secondary atom site location: difference Fourier map
*R*[*F*^2^ > 2σ(*F*^2^)] = 0.030	Hydrogen site location: inferred from neighbouring sites
*wR*(*F*^2^) = 0.075	H-atom parameters constrained
*S* = 1.06	*w* = 1/[σ^2^(*F*_o_^2^) + (0.0258*P*)^2^ + 0.4345*P*] where *P* = (*F*_o_^2^ + 2*F*_c_^2^)/3
4031 reflections	(Δ/σ)_max_ = 0.001
227 parameters	Δρ_max_ = 0.49 e Å^−^^3^
0 restraints	Δρ_min_ = −0.40 e Å^−^^3^

### Special details

**Table:**

Geometry. All s.u.'s (except the s.u. in the dihedral angle between two l.s. planes) are estimated using the full covariance matrix. The cell s.u.'s are taken into account individually in the estimation of s.u.'s in distances, angles and torsion angles; correlations between s.u.'s in cell parameters are only used when they are defined by crystal symmetry. An approximate (isotropic) treatment of cell s.u.'s is used for estimating s.u.'s involving l.s. planes.
Refinement. Refinement of *F*^2^ against ALL reflections. The weighted *R*-factor w*R* and goodness of fit *S* are based on *F*^2^, conventional *R*-factors *R* are based on *F*, with *F* set to zero for negative *F*^2^. The threshold expression of *F*^2^ > 2σ(*F*^2^) is used only for calculating *R*-factors(gt) etc. and is not relevant to the choice of reflections for refinement. *R*-factors based on *F*^2^ are statistically about twice as large as those based on *F*, and *R*-factors based on ALL data will be even larger.

### Fractional atomic coordinates and isotropic or equivalent isotropic displacement parameters (Å^2^)

**Table:**

	*x*	*y*	*z*	*U*_iso_*/*U*_eq_	
Br1	0.57980 (3)	0.18645 (3)	0.314671 (17)	0.03502 (8)	
S1	0.84031 (6)	0.35008 (6)	0.82867 (4)	0.02420 (11)	
F1	0.3382 (2)	−0.0623 (2)	0.89171 (12)	0.0539 (4)	
O1	0.90963 (19)	0.80950 (18)	0.64831 (11)	0.0290 (3)	
O2	0.92123 (19)	0.21364 (19)	0.79427 (12)	0.0314 (3)	
O3	0.90169 (19)	0.3885 (2)	0.91876 (11)	0.0325 (3)	
C1	0.8517 (2)	0.5360 (2)	0.72789 (15)	0.0234 (4)	
C2	0.8204 (2)	0.5642 (2)	0.61823 (15)	0.0225 (4)	
C3	0.7631 (2)	0.4684 (2)	0.55178 (15)	0.0215 (4)	
C4	0.7185 (3)	0.2941 (2)	0.58317 (15)	0.0261 (4)	
H4	0.7272	0.2323	0.6538	0.031*	
C5	0.6630 (3)	0.2119 (3)	0.51466 (16)	0.0275 (4)	
H5	0.6325	0.0948	0.5376	0.033*	
C6	0.6516 (2)	0.3023 (3)	0.41023 (16)	0.0253 (4)	
C7	0.6938 (3)	0.4703 (3)	0.37573 (16)	0.0270 (4)	
H7	0.6852	0.5286	0.3045	0.032*	
C8	0.7502 (2)	0.5582 (2)	0.44463 (15)	0.0234 (4)	
C9	0.7940 (3)	0.7335 (3)	0.40720 (16)	0.0274 (4)	
H9	0.7845	0.7893	0.3357	0.033*	
C10	0.8488 (3)	0.8229 (3)	0.47063 (16)	0.0276 (4)	
H10	0.8790	0.9399	0.4456	0.033*	
C11	0.8588 (2)	0.7340 (2)	0.57511 (16)	0.0247 (4)	
C12	0.9047 (3)	0.6874 (3)	0.74099 (16)	0.0277 (4)	
C13	0.9565 (3)	0.7507 (3)	0.82888 (19)	0.0394 (5)	
H13A	1.0769	0.7176	0.8436	0.059*	
H13B	0.8801	0.7012	0.8906	0.059*	
H13C	0.9467	0.8751	0.8104	0.059*	
C14	0.6173 (2)	0.2986 (3)	0.85656 (14)	0.0238 (4)	
C15	0.5063 (3)	0.4128 (3)	0.88799 (16)	0.0289 (4)	
H15	0.5463	0.5223	0.8879	0.035*	
C16	0.3356 (3)	0.3642 (3)	0.91954 (17)	0.0354 (5)	
H16	0.2575	0.4416	0.9404	0.043*	
C17	0.2788 (3)	0.2043 (3)	0.92085 (17)	0.0363 (5)	
H17	0.1623	0.1704	0.9431	0.044*	
C18	0.3928 (3)	0.0953 (3)	0.88949 (17)	0.0349 (5)	
C19	0.5630 (3)	0.1383 (3)	0.85604 (16)	0.0298 (4)	
H19	0.6394	0.0612	0.8336	0.036*	

### Atomic displacement parameters (Å^2^)

**Table:**

	*U*^11^	*U*^22^	*U*^33^	*U*^12^	*U*^13^	*U*^23^
Br1	0.04802 (14)	0.03053 (13)	0.02960 (12)	−0.00014 (9)	−0.00906 (9)	−0.01191 (9)
S1	0.0227 (2)	0.0285 (3)	0.0205 (2)	0.00142 (18)	−0.00143 (18)	−0.00511 (19)
F1	0.0640 (10)	0.0522 (9)	0.0498 (9)	−0.0291 (8)	0.0038 (8)	−0.0209 (7)
O1	0.0344 (8)	0.0268 (7)	0.0263 (7)	−0.0069 (6)	0.0010 (6)	−0.0084 (6)
O2	0.0297 (7)	0.0319 (8)	0.0309 (8)	0.0074 (6)	−0.0006 (6)	−0.0064 (6)
O3	0.0311 (7)	0.0439 (9)	0.0238 (7)	−0.0008 (7)	−0.0066 (6)	−0.0101 (7)
C1	0.0226 (9)	0.0264 (10)	0.0206 (9)	−0.0019 (7)	0.0018 (7)	−0.0061 (8)
C2	0.0218 (9)	0.0231 (9)	0.0219 (9)	0.0006 (7)	0.0024 (7)	−0.0055 (7)
C3	0.0180 (8)	0.0241 (9)	0.0217 (9)	0.0011 (7)	0.0024 (7)	−0.0056 (7)
C4	0.0311 (10)	0.0240 (10)	0.0215 (9)	0.0004 (8)	0.0007 (8)	−0.0039 (8)
C5	0.0335 (10)	0.0218 (10)	0.0264 (10)	−0.0009 (8)	−0.0005 (8)	−0.0052 (8)
C6	0.0244 (9)	0.0295 (10)	0.0245 (10)	0.0013 (8)	−0.0028 (8)	−0.0114 (8)
C7	0.0288 (10)	0.0285 (10)	0.0223 (10)	0.0029 (8)	−0.0028 (8)	−0.0044 (8)
C8	0.0220 (9)	0.0249 (10)	0.0219 (9)	0.0014 (7)	0.0005 (7)	−0.0047 (8)
C9	0.0302 (10)	0.0261 (10)	0.0224 (10)	0.0008 (8)	−0.0013 (8)	−0.0007 (8)
C10	0.0286 (10)	0.0228 (10)	0.0288 (10)	−0.0038 (8)	0.0022 (8)	−0.0030 (8)
C11	0.0233 (9)	0.0248 (10)	0.0263 (10)	−0.0028 (7)	0.0017 (8)	−0.0081 (8)
C12	0.0275 (10)	0.0310 (11)	0.0249 (10)	−0.0034 (8)	0.0034 (8)	−0.0090 (8)
C13	0.0503 (14)	0.0389 (13)	0.0328 (12)	−0.0120 (11)	−0.0007 (10)	−0.0160 (10)
C14	0.0245 (9)	0.0287 (10)	0.0161 (9)	−0.0011 (8)	−0.0013 (7)	−0.0021 (7)
C15	0.0287 (10)	0.0309 (11)	0.0250 (10)	0.0022 (8)	−0.0013 (8)	−0.0041 (8)
C16	0.0276 (10)	0.0470 (14)	0.0281 (11)	0.0073 (10)	0.0001 (9)	−0.0050 (10)
C17	0.0250 (10)	0.0559 (15)	0.0252 (11)	−0.0068 (10)	−0.0041 (8)	−0.0053 (10)
C18	0.0413 (12)	0.0402 (12)	0.0224 (10)	−0.0150 (10)	−0.0042 (9)	−0.0056 (9)
C19	0.0363 (11)	0.0321 (11)	0.0209 (10)	−0.0009 (9)	−0.0005 (8)	−0.0075 (8)

### Geometric parameters (Å, º)

**Table:**

Br1—C6	1.8986 (19)	C7—H7	0.9500
S1—O2	1.4358 (15)	C8—C9	1.421 (3)
S1—O3	1.4363 (15)	C9—C10	1.351 (3)
S1—C1	1.736 (2)	C9—H9	0.9500
S1—C14	1.770 (2)	C10—C11	1.398 (3)
F1—C18	1.352 (3)	C10—H10	0.9500
O1—C12	1.365 (3)	C12—C13	1.487 (3)
O1—C11	1.371 (2)	C13—H13A	0.9800
C1—C12	1.367 (3)	C13—H13B	0.9800
C1—C2	1.460 (3)	C13—H13C	0.9800
C2—C11	1.378 (3)	C14—C19	1.382 (3)
C2—C3	1.428 (3)	C14—C15	1.386 (3)
C3—C4	1.412 (3)	C15—C16	1.388 (3)
C3—C8	1.434 (3)	C15—H15	0.9500
C4—C5	1.367 (3)	C16—C17	1.379 (3)
C4—H4	0.9500	C16—H16	0.9500
C5—C6	1.402 (3)	C17—C18	1.369 (3)
C5—H5	0.9500	C17—H17	0.9500
C6—C7	1.361 (3)	C18—C19	1.380 (3)
C7—C8	1.406 (3)	C19—H19	0.9500
			
O2—S1—O3	118.58 (9)	C9—C10—C11	116.43 (19)
O2—S1—C1	109.57 (9)	C9—C10—H10	121.8
O3—S1—C1	107.58 (9)	C11—C10—H10	121.8
O2—S1—C14	107.07 (9)	O1—C11—C2	111.44 (17)
O3—S1—C14	106.73 (9)	O1—C11—C10	122.61 (18)
C1—S1—C14	106.70 (9)	C2—C11—C10	125.94 (19)
C12—O1—C11	107.20 (15)	O1—C12—C1	109.96 (17)
C12—C1—C2	107.40 (17)	O1—C12—C13	113.53 (18)
C12—C1—S1	122.42 (15)	C1—C12—C13	136.5 (2)
C2—C1—S1	130.10 (15)	C12—C13—H13A	109.5
C11—C2—C3	118.08 (18)	C12—C13—H13B	109.5
C11—C2—C1	104.00 (17)	H13A—C13—H13B	109.5
C3—C2—C1	137.92 (18)	C12—C13—H13C	109.5
C4—C3—C2	125.37 (18)	H13A—C13—H13C	109.5
C4—C3—C8	117.97 (18)	H13B—C13—H13C	109.5
C2—C3—C8	116.66 (17)	C19—C14—C15	121.94 (19)
C5—C4—C3	121.73 (18)	C19—C14—S1	118.52 (15)
C5—C4—H4	119.1	C15—C14—S1	119.23 (16)
C3—C4—H4	119.1	C14—C15—C16	118.7 (2)
C4—C5—C6	119.28 (19)	C14—C15—H15	120.6
C4—C5—H5	120.4	C16—C15—H15	120.6
C6—C5—H5	120.4	C17—C16—C15	120.5 (2)
C7—C6—C5	121.39 (18)	C17—C16—H16	119.8
C7—C6—Br1	119.44 (15)	C15—C16—H16	119.8
C5—C6—Br1	119.15 (15)	C18—C17—C16	118.9 (2)
C6—C7—C8	120.53 (18)	C18—C17—H17	120.5
C6—C7—H7	119.7	C16—C17—H17	120.5
C8—C7—H7	119.7	F1—C18—C17	119.1 (2)
C7—C8—C9	119.75 (18)	F1—C18—C19	118.0 (2)
C7—C8—C3	119.09 (18)	C17—C18—C19	122.9 (2)
C9—C8—C3	121.16 (18)	C18—C19—C14	117.1 (2)
C10—C9—C8	121.72 (19)	C18—C19—H19	121.5
C10—C9—H9	119.1	C14—C19—H19	121.5
C8—C9—H9	119.1		
			
O2—S1—C1—C12	−132.60 (17)	C12—O1—C11—C2	0.1 (2)
O3—S1—C1—C12	−2.4 (2)	C12—O1—C11—C10	179.98 (18)
C14—S1—C1—C12	111.81 (17)	C3—C2—C11—O1	179.17 (16)
O2—S1—C1—C2	43.6 (2)	C1—C2—C11—O1	−0.1 (2)
O3—S1—C1—C2	173.78 (17)	C3—C2—C11—C10	−0.7 (3)
C14—S1—C1—C2	−72.00 (19)	C1—C2—C11—C10	−179.99 (19)
C12—C1—C2—C11	0.1 (2)	C9—C10—C11—O1	−178.91 (18)
S1—C1—C2—C11	−176.57 (15)	C9—C10—C11—C2	1.0 (3)
C12—C1—C2—C3	−178.9 (2)	C11—O1—C12—C1	0.0 (2)
S1—C1—C2—C3	4.4 (3)	C11—O1—C12—C13	−179.86 (17)
C11—C2—C3—C4	−179.95 (18)	C2—C1—C12—O1	0.0 (2)
C1—C2—C3—C4	−1.0 (4)	S1—C1—C12—O1	176.92 (13)
C11—C2—C3—C8	0.0 (3)	C2—C1—C12—C13	179.8 (2)
C1—C2—C3—C8	178.9 (2)	S1—C1—C12—C13	−3.3 (4)
C2—C3—C4—C5	179.37 (18)	O2—S1—C14—C19	8.63 (18)
C8—C3—C4—C5	−0.5 (3)	O3—S1—C14—C19	−119.31 (16)
C3—C4—C5—C6	0.6 (3)	C1—S1—C14—C19	125.89 (16)
C4—C5—C6—C7	−0.3 (3)	O2—S1—C14—C15	−177.62 (15)
C4—C5—C6—Br1	178.52 (15)	O3—S1—C14—C15	54.44 (18)
C5—C6—C7—C8	−0.1 (3)	C1—S1—C14—C15	−60.36 (18)
Br1—C6—C7—C8	−178.92 (14)	C19—C14—C15—C16	−0.1 (3)
C6—C7—C8—C9	179.91 (18)	S1—C14—C15—C16	−173.67 (16)
C6—C7—C8—C3	0.2 (3)	C14—C15—C16—C17	0.9 (3)
C4—C3—C8—C7	0.1 (3)	C15—C16—C17—C18	−0.7 (3)
C2—C3—C8—C7	−179.79 (17)	C16—C17—C18—F1	179.29 (19)
C4—C3—C8—C9	−179.58 (17)	C16—C17—C18—C19	−0.3 (3)
C2—C3—C8—C9	0.5 (3)	F1—C18—C19—C14	−178.59 (18)
C7—C8—C9—C10	−179.96 (19)	C17—C18—C19—C14	1.0 (3)
C3—C8—C9—C10	−0.2 (3)	C15—C14—C19—C18	−0.7 (3)
C8—C9—C10—C11	−0.5 (3)	S1—C14—C19—C18	172.82 (15)

## References

[bb1] Brandenburg, K. (1998). *DIAMOND* Crystal Impact GbR, Bonn, Germany.

[bb2] Bruker (2009). *APEX2*, *SADABS* and *SAINT* Bruker AXS Inc., Madison, Wisconsin, USA.

[bb3] Choi, H. D., Seo, P. J., Son, B. W. & Lee, U. (2008). *Acta Cryst.* E**64**, o1158. 10.1107/S1600536808015286 PMC2961428 21202666

[bb4] Choi, H. D., Seo, P. J., Son, B. W. & Lee, U. (2011). *Acta Cryst.* E**67**, o280. 10.1107/S160053681005436X PMC3051741 21522972

[bb5] Farrugia, L. J. (2012). *J. Appl. Cryst.* **45**, 849–854.

[bb6] Sheldrick, G. M. (2008). *Acta Cryst.* A**64**, 112–122. 10.1107/S0108767307043930 18156677

